# A Twin Study of Early-Childhood Asthma in Puerto Ricans

**DOI:** 10.1371/journal.pone.0068473

**Published:** 2013-07-03

**Authors:** Supinda Bunyavanich, Judy L. Silberg, Jessica Lasky-Su, Nathan A. Gillespie, Nancy E. Lange, Glorisa Canino, Juan C. Celedόn

**Affiliations:** 1 Department of Pediatrics, Icahn School of Medicine at Mount Sinai, New York, New York, United States of America; 2 Department of Genetics and Genomic Sciences, Icahn School of Medicine at Mount Sinai, New York, New York, United States of America; 3 Mindich Child Health and Development Institute, Icahn School of Medicine at Mount Sinai, New York, New York, United States of America; 4 Department of Medicine, Brigham and Women's Hospital, Boston, Massachusetts, United States of America; 5 Harvard Medical School, Boston, Massachusetts, United States of America; 6 Department of Human and Molecular Genetics, Virginia Commonwealth University, Richmond, Virginia, United States of America; 7 Virginia Institute for Psychiatric and Behavioral Genetics, Virginia Commonwealth University, Richmond, Virginia, United States of America; 8 Queensland Institute of Medical Research, Herston, Queensland, Australia; 9 Behavioral Sciences Research Institute, University of Puerto Rico Medical School, San Juan, Puerto Rico; 10 Division of Pulmonary Medicine, Allergy, and Immunology, Children's Hospital of Pittsburgh of the University of Pittsburgh Medical Center, Pittsburgh, Pennsylvania, United States of America; 11 Departments of Pediatrics and Internal Medicine, University of Pittsburgh School of Medicine, Pittsburgh, Pennsylvania, United States of America; 12 Department of Human Genetics, University of Pittsburgh Graduate School of Public Health, Pittsburgh, Pennsylvania, United States of America; Yale School of Public Health, United States of America

## Abstract

**Background:**

The relative contributions of genetics and environment to asthma in Hispanics or to asthma in children younger than 3 years are not well understood.

**Objective:**

To examine the relative contributions of genetics and environment to early-childhood asthma by performing a longitudinal twin study of asthma in Puerto Rican children ≤3 years old.

**Methods:**

678 twin infants from the Puerto Rico Neo-Natal Twin Registry were assessed for asthma at age 1 year, with follow-up data obtained for 624 twins at age 3 years. Zygosity was determined by DNA microsatellite profiling. Structural equation modeling was performed for three phenotypes at ages 1 and 3 years: physician-diagnosed asthma, asthma medication use in the past year, and ≥1 hospitalization for asthma in the past year. Models were additionally adjusted for early-life environmental tobacco smoke exposure, sex, and age.

**Results:**

The prevalences of physician-diagnosed asthma, asthma medication use, and hospitalization for asthma were 11.6%, 10.8%, 4.9% at age 1 year, and 34.1%, 40.1%, and 8.5% at 3 years, respectively. Shared environmental effects contributed to the majority of variance in susceptibility to physician-diagnosed asthma and asthma medication use in the first year of life (84%–86%), while genetic effects drove variance in all phenotypes (45%–65%) at age 3 years. Early-life environmental tobacco smoke, sex, and age contributed to variance in susceptibility.

**Conclusion:**

Our longitudinal study in Puerto Rican twins demonstrates a changing contribution of shared environmental effects to liability for physician-diagnosed asthma and asthma medication use between ages 1 and 3 years. Early-life environmental tobacco smoke reduction could markedly reduce asthma morbidity in young Puerto Rican children.

## Introduction

Twin studies offer a unique opportunity to examine the relative contributions of genetic and environmental factors to childhood asthma. Monozygotic twins completely share their genetics and early-life environment, while dizygotic twins share only half their genetics and all their early-life environment. Early twin studies of asthma concluded that the environment exerted stronger effects than genetics.[1], [2] These findings were consistent with epidemiologic studies demonstrating a role for environmental factors such as tobacco smoke in asthma pathogenesis.[3], [4]

Recent twin studies of asthma argue that genetic factors primarily explain asthma susceptibility,[5]–[7] and that shared environmental factors do not contribute to its variation.[8] In this study, we assess the relative contributions of genetics and environment to asthma susceptibility and its variation with age by conducting a longitudinal twin study of asthma starting in infancy. To our knowledge, this is the first twin study to examine asthma at such an early age. [8]–[13]

Most twin studies of asthma have been conducted in Caucasian adults.[14] Since candidate gene and genome-wide association studies have demonstrated ethnic-specific genetic effects for asthma (e.g. in Hispanic subgroups),[15], [16] and studies of environmental risk factors for asthma have also identified ethnic-specific results,[17] twin studies could also yield ethnic-specific findings for the genetic and environmental contributions to childhood asthma. We report the first twin study of asthma in Puerto Ricans, a Hispanic subgroup with the highest prevalence of childhood asthma among U.S. ethnic groups.[18]–[20]

## Methods

### Population

**Figure 1:**
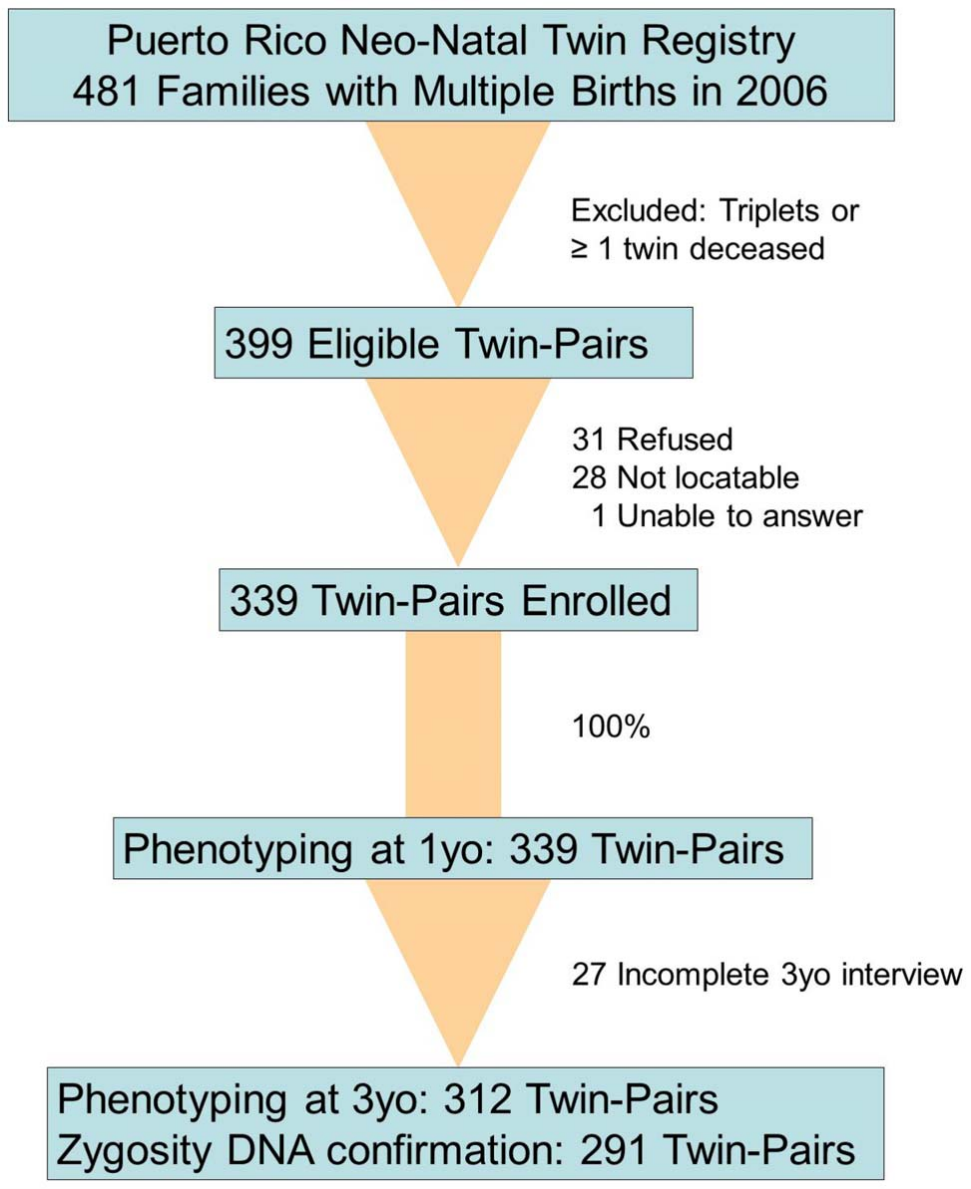
Study design of the Puerto Rico Neo-Natal Twin Registry.

All families with multiple gestation pregnancy in Puerto Rico in 2006 were considered for inclusion. Contact information was obtained from the Puerto Rico Neonatal Twin Registry with assistance from the Puerto Rico Department of Health. Families were contacted within 3 months of birth and were not selected for any disease. Of the 481 families with multiple births, 82 were ineligible (**Figure 1**). Of the 399 eligible families, 339 (85%) families with twins elected to participate. At age 1 year, parents answered questionnaires addressing demographics, asthma, *in utero* smoke (IUS) exposure, and environmental tobacco smoke (ETS). Parents were recontacted when the children were age 3 years, with 312 (92%) of the original 339 families completing follow-up questionnaires. Questions regarding ETS exposure were not repeated at age 3 years. Written informed consent was obtained from all study participants, including parents on the behalf of children participants. The study was approved by the Institutional Review Boards of the University of Puerto Rico and Virginia Commonwealth University (IRB#4774).

### Outcomes

For each twin, outcomes assessed at 1 and 3 years of age included parental report of physician-diagnosed asthma, asthma medication use in the past year, and any hospitalization for asthma in the past year. Specifically, study staff asked parents, “Has (name of child) ever been diagnosed by a doctor as having asthma.” Asthma medication use in the past year was classified as positive if a parent answered yes to either of these two questions: “Has (name of child) used any prescribed asthma medications in the last 12 months,” and “During the last 12 months, has (name of child) had any oral steroids (eg. prednisone burst, prelone)?” A child was considered to have had a hospitalization for asthma in the past year if a parent answered yes to “During the last 12 months, has (name of child) been hospitalized for asthma?”

### Zygosity

Zygosity was assessed by questionnaire and DNA testing. Parents were asked if their twins were identical or fraternal. Although self-reported zygosity has been shown to be adequate in 95–98% of cases,[21] we sought to maximize the accuracy of our zygosity classification with DNA testing. We collected saliva from 292 participating twin pairs (584 subjects) for DNA extraction and genotyping of 47 independent microsatellite markers. Subjects with uniformly concordant microsatellite calls were considered monozygotic by DNA testing. The agreement rate between parental-reported zygosity and DNA testing was assessed using the Kappa statistic and measures of sensitivity and specificity. The DNA results served as our gold-standard for zygosity determination.

### Statistical Analysis

Probandwise concordance rates were calculated for monozygotic and dizygotic twins for each phenotype according to standard methods.[22]

**Figure 2:**
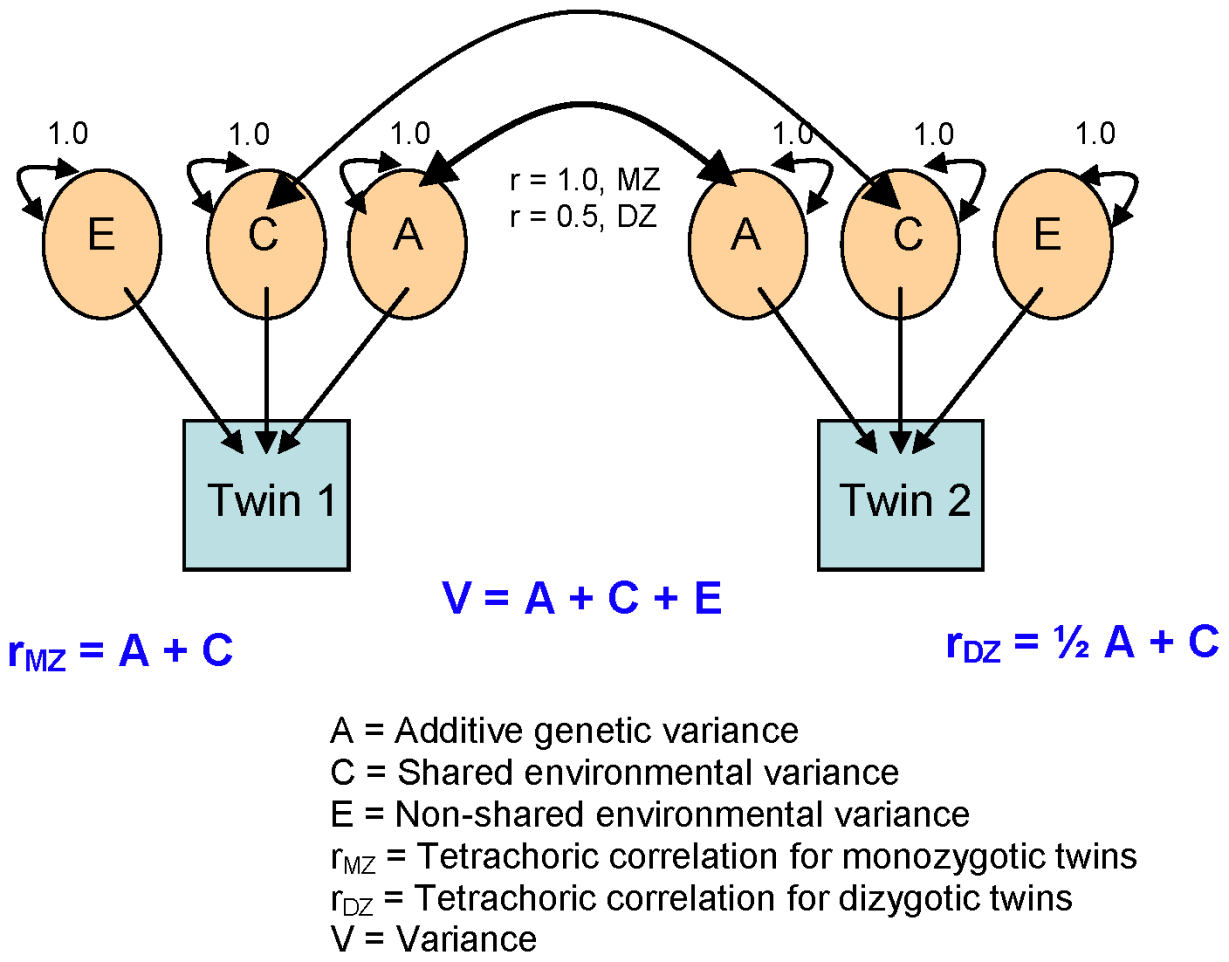
ACE model for calculating genetic and environmental influence from twin correlations.[**23**]

To estimate the contributions of genetic and environmental risk factors to susceptibility to asthma phenotypes at ages 1 and 3 years, we fitted structural equation models to estimate variance components using Mx (**Figure 2**).[23] Mx is a matrix algebra interpreter and numerical optimizer frequently used in the analysis of twin data. Standard models within twin research assume that susceptibility to outcomes are a linear function of additive genetic effects [A], shared environmental effects [C], and nonshared random environmental effects [E].[23] The tetrachoric correlation is used in twin analyses as a standard measure of correlation or agreement between binary variables. The tetrachoric correlation among monozygotic twins (rMZ)  = A+C, and the tetrachoric correlation among dizygotic twins (rDZ)  = ½ A+C.[23]

Based on covariances estimated from tetrachoric correlations, univariate models were created with A, C, and E latent sources of variance.[23] To assess for possible effect modification by sex, we created sex-stratified models (allowing for different male and female estimates) for each phenotype and compared them by likelihood ratio χ^2^ test to the corresponding unstratified model. Models incorporating covariates for sex, age, and early-life ETS were then created. These adjusted models were compared to univariate models by likelihood ratio χ^2^ tests to decipher potential contributors to detected environmental variances. These covariates were chosen *a priori* given previous epidemiologic data. Specifically, male sex and younger age are known risk factors for asthma in early childhood.[24], [25] With regard to ETS, (1) Puerto Rican women have the highest rate of perinatal smoking among all U.S. Hispanics (9.2% prevalence),[26] (2) ETS demonstrated a significant effect on hospitalizations for asthma at age 1 year in this cohort,[27] and (3) IUS has been associated with asthma in Puerto Ricans.[28] Early-life ETS exposure was defined as either IUS or ETS exposure in the first year.

## Results

The phenotypic characteristics of children in the Puerto Rico Neonatal Twin Registry at ages 1 and 3 years are shown in **Table 1**. Although the rates of zygosity by parental report and DNA testing were equal, this did not result from identical zygosity calls for each twin pair between the methods. There was 88.3% agreement between parental-reported zygosity and DNA testing (Kappa statistic 0.74 (95% CI = 0.66−0.82)). Parental-reported zygosity had 82.5% sensitivity and 91.5% specificity for zygosity by DNA testing. There was no significant difference in socioeconomic status between monozygotic and dizygotic twin pairs.

**Table 1:** Characteristics of participants at ages 1 and 3 years.

Characteristic	Age 1 year (n = 678)	Age 3 years (n = 624)
Female	352 (51.9%)	328 (52.6%)
Monozygotic	240 (35.4%)	206 (35.3%)*
Physician-diagnosed asthma	78 (11.6%)	213 (34.1%)
Asthma medication use in past 12 months	73 (10.8%)	250 (40.1%)
Hospitalized for asthma in past 12 months	33 (4.9%)	53 (8.5%)
Intrauterine smoke exposure	20 (3%)	
Environmental tobacco smoke exposure∧	94 (13.9%)	

Values are reported as number (%) or mean (SD).

*By DNA testing available for 584 subjects (292 twin pairs)); prevalence per parental report for 678 subjects at 1yo was the same (240 (35.4%)).

∧Defined as cigarette smoke exposure ≥2 times/week in first year of life.

A substantial minority of twins experienced early-life ETS (**Table 1**). From age 1 year to age 3 years, the prevalence of physician-diagnosed asthma nearly tripled, asthma medication use in the past year nearly quadrupled, and the prevalence of ≥1 hospitalization for asthma in the past year nearly doubled. The proportions of children who had each of the outcomes of interest at both age 1 year and age 3 years were 8.8% for physician-diagnosed asthma, 7.5% for asthma medication use in the past year, and 1.3% for ≥1 hospitalization for asthma in the past year.

**Table 2** shows the prevalence and resemblance between twins for asthma-related phenotypes at age 1 year. Probandwise concordance was slightly lower for dizygotic subjects relative to monozygotic subjects for each of these phenotypes. Accordingly, tetrachoric correlations were lower for dizygotic subjects.

**Table 2:** Prevalence and resemblance between twins for asthma-related phenotypes at age 1 year.

Phenotype	Pairs*	Cases	Discordant pairs	Concordant pairs	Probandwise concordance	Tetrachoric correlation (95% CI)
Physician-diagnosed asthma						
MZ	101	24	4	97	0.980	0.97 (0.91–1.00)
DZ	188	43	12	176	0.967	0.92 (0.84–1.00)
Asthma medications use in past 12 months						
MZ	103	25	5	98	0.975	0.96 (0.88–1.00)
DZ	189	38	13	176	0.964	0.89 (0.77–1.00)
Hospitalized for asthma in past 12 months						
MZ	102	13	3	99	0.985	0.96 (0.87–1.00)
DZ	187	17	13	174	0.964	0.53 (0.11–0.96)

MZ =  monozygotic, DZ =  dizygotic.

*584 subjects with zygosity confirmed by DNA testing were used in this analysis.

**Table 3** shows the prevalence and resemblance between twins for asthma-related phenotypes at age 3 years. Probandwise concordance and tetrachoric correlations were again lower for dizygotic subjects compared to monozygotic subjects for each of these phenotypes.

**Table 3:** Prevalence and resemblance between twins for asthma-related phenotypes at age 3 years.

Phenotype	Pairs*	Cases	Discordant pairs	Concordant pairs	Probandwise concordance	Tetrachoric correlation (95% CI)
Physician-diagnosed asthma						
MZ	102	84	10	92	0.948	0.95 (0.90–1.00)
DZ	189	117	49	140	0.851	0.61 (0.43–0.78)
Asthma medications use in past 12 months						
MZ	102	90	10	92	0.948	0.95 (0.80–1.00)
DZ	189	144	48	141	0.855	0.67 (0.52–0.82)
Hospitalized for asthma in past 12 months						
MZ	102	23	7	95	0.964	0.91 (0.78–1.00)
DZ	189	29	19	170	0.947	0.58 (0.28–0.88)

MZ =  monozygotic, DZ =  dizygotic.

*584 subjects with zygosity confirmed by DNA testing were used in this analysis.

Variance components from structural equation modeling of asthma-related phenotypes at age 1 year are shown in **Table 4**. Genetic factors accounted for 11% (A), and shared environmental effects accounted for 84% to 86% (C) of the variance in susceptibility to physician-diagnosed asthma and asthma medication use in the past year. There was no significant effect of sex stratification on model fit for any phenotype at age 1 year (**Table S1 in File S1**). Sex-adjusted models for physician-diagnosed asthma and asthma medication use were not significantly different from the corresponding unadjusted models (**Table 4**). However, models for asthma medication use that were additionally adjusted for early-life ETS and age significantly differed from unadjusted models (P value <0.001), suggesting that early-life ETS and age may contribute to shared environmental effects in asthma medication use. Early-life ETS exposure and older age were associated with asthma medication use, and older age was associated with physician-diagnosed asthma.

**Table 4:** Variance component analysis of asthma-related phenotypes at age 1 year.

Phenotype	A# (95% CI)	C (95% CI)	E (95% CI)	P value*
Physician-diagnosed asthma	0.11 (0.00–0.37)	0.86 (0.60–0.97)	0.03 (0.003–0.13)	
Adjusted for sex	0.09 (0.00–0.34)	0.88 (0.64–0.98)	0.03 (0.003–0.13)	0.06
Adjusted for sex and early-life ETS∧	0.09 (0.00–0.34)	0.88 (0.64–0.98)	0.03 (0.003–0.13)	0.06
Adjusted for sex and age	0.11 (0.11–0.12)	0.86 (0.83–0.86)	0.03 (0.03–0.05)	**<0.001**
Asthma medication use in past 12 months	0.11 (0.00–0.43)	0.84 (0.54–0.97)	0.05 (0.01–0.16)	
Adjusted for sex	0.09 (0.00–0.39)	0.86 (0.58–0.97)	0.05 (0.01–0.16)	0.12
Adjusted for sex and early-life ETS	0.09 (0.00–0.40)	0.86 (0.56–0.97)	0.05 (0.01–0.17)	**<0.001**
Adjusted for sex and age	0.11 (0.11–0.14)	0.84 (0.78–0.84)	0.05 (0.05–0.09)	**<0.001**
Hospitalized for asthma in past 12 months	0.95 (0.12–0.99)	0.0004 (0.00–0.74)	0.05 (0.003–0.26)	
Adjusted for sex	0.55 (0.00–0.99)	0.40 (0.00–0.91)	0.05 (0.004–0.28)	**<0.001**
Adjusted for sex and early-life ETS	0.59 (0.00–0.99)	0.35 (0.00–0.90)	0.06 (0.004–0.31)	**<0.001**
Adjusted for sex and age	0.70 (0.001–0.99)	0.24 (0.00–0.24)	0.06 (0.06–0.11)	**<0.001**

584 subjects with zygosity confirmed by DNA testing were used in these models.

# A =  additive genetic variance, C =  shared environmental variance, E =  non-shared environmental variance [23].

*P-value for likelihood ratio χ2 test comparing adjusted to univariate model.

∧Early-life environmental tobacco smoke (ETS) defined as intrauterine smoke exposure or cigarette exposure ≥2×/week during the first year of life.

In contrast to our findings for physician-diagnosed asthma and asthma medication use, genetic factors accounted for almost all of variance in susceptibility to hospitalization for asthma among all twins (A = 0.95). Notwithstanding, multivariate models with sex, early-life ETS, and age adjustments yielded models with significantly better fit and higher C estimates, supporting that sex, early-life ETS, and age all contribute to shared environmental effect in variance to susceptibility to hospitalizations for asthma.

Variance components from structural equation modeling of asthma-related phenotypes at age 3 years are shown in **Table 5**. In contrast to the results at age 1 year, genetic factors accounted for a substantial proportion (45%–65%) of the unadjusted variance in susceptibility to all three phenotypes at age 3 years. There was no significant effect of sex stratification on model fit for any phenotype at age 3 years (**Table S2 in File S1**). Adjustments for sex and age resulted in models with better fit for all phenotypes (**Table 5**), with male sex and older age positively associated with the outcomes. Adjustment for early-life ETS did not significantly alter model fit. The substantial genetic contribution to variance in susceptibility to all three phenotypes remained evident in the adjusted models and ranged from 34% to 59%.

**Table 5:** Variance component analysis of asthma-related phenotypes at age 3 years.

Phenotype	A#(95% CI)	C (95% CI)	E (95% CI)	P value*
Physician-diagnosed asthma	0.65 (0.31–0.97)	0.29 (0.00–0.59)	0.06 (0.02–0.16)	
Adjusted for sex	0.57 (0.24–0.96)	0.37 (0.00–0.66)	0.06 (0.02–0.16)	**<0.001**
Adjusted for sex and early-life ETS∧	0.57 (0.23–0.96)	0.37 (0.00–0.66)	0.06 (0.02–0.16)	0.11
Adjusted for sex and age	0.59 (0.59–0.61)	0.35 (0.27–0.35)	0.06 (0.06–0.12)	**0.03**
Asthma Medications in past 12 months	0.45 (0.15–0.80)	0.52 (0.17–0.77)	0.03 (0.005–0.14)	
Adjusted for sex	0.34 (0.05–0.66)	0.63 (0.31–0.85)	0.03 (0.005–0.15)	**<0.001**
Adjusted for sex and early-life ETS∧	0.34 (0.05–0.67)	0.62 (0.31–0.85)	0.04 (0.005–0.15)	0.23
Adjusted for sex and age	0.37 (0.37–0.37)	0.60 (0.57–0.60)	0.04 (0.04–0.04)	**<0.001**
Hospitalized for asthma in past 12 months	0.56 (0.00–0.97)	0.33 (0.00–0.82)	0.11 (0.02–0.33)	
Adjusted for sex	0.57 (0.00–0.97)	0.32 (0.00–0.82)	0.11 (0.02–0.33)	0.09
Adjusted for sex and early-life ETS∧	0.59 (0.00–0.97)	0.30 (0.00–0.81)	0.11 (0.03–0.32)	0.10
Adjusted for sex and age	0.59 (0.00–0.97)	0.30 (0.00–0.91)	0.11 (0.03–0.32)	**0.01**

584 subjects with zygosity confirmed by DNA testing were used in these models.

# A =  additive genetic variance, C =  shared environmental variance, E =  non-shared environmental variance.[23].

*P-value for likelihood ratio χ2 test comparing adjusted model to univariate model.

∧Early-life environmental tobacco smoke (ETS) defined as intrauterine smoke exposure or cigarette exposure ≥2×/week during the first year of life.

Structural equation models of asthma-related phenotypes persisting between ages 1 and 3 years (e.g. reported asthma medication use at both ages) were unstable due to low prevalence of these outcomes; the results from these particular models are therefore not shown.

## Discussion

The diagnosis of asthma is challenging before age 6 years.[29] Although wheeze occurs in approximately one third of children before age 3 years, ∼59% of children who wheeze before age 3 years will have no wheeze by age 6 years (“transient wheeze”).[25] Because it is difficult to predict whether a wheezing infant will have transient vs. persistent wheeze,[30] physicians may be less likely to diagnose asthma at age 1 year than at age 3 years. This would partly explain our finding of a higher prevalence of asthma-related outcomes at age 3 years than at age 1 year.

A large proportion of children diagnosed with asthma or receiving medications for asthma at age 1 year may have transient wheeze. Early-life ETS and viral infection are risk factors for transient wheeze,[25], [30] and these exposures would be shared between twins. This would explain why we observed that shared environmental effects contributed ∼85% to susceptibility to physician-diagnosed asthma and asthma medication use in the first year of life.

In contrast, physician-diagnosed asthma or asthma medication use at age 3 years may be less likely to be due to viral illnesses and more likely to represent “true asthma”. Risk factors for wheeze persisting at or after age 3 years include atopy and a history of maternal asthma, [25] which have a recognized genetic basis. Consistent with this, we observed that physician-diagnosed asthma had a more significant genetic component at age 3 years than at age 1 year.

Unlike physician-diagnosed asthma or asthma medication use, a hospitalization for asthma signals a more severe manifestation of asthma. Severe asthma may represent a phenotype distinct from mild or moderate asthma.[31] and often manifests in children in the first 24 months of life.[30] Severe asthma has been associated with more symptoms, greater airway obstruction, increased bronchial responsiveness to methacholine, higher F(ENO) and greater sensitization to aeroallergens.[31] These risk factors reflect intrinsic characteristics that are more likely to be genetically rather than environmentally determined. The relative age-independency of our findings for a genetic contribution to asthma-related hospitalizations is consistent with these characteristics of severe asthma.

Our results suggest that ETS (an exposure that would be shared by twins in a given pair) partly explains the estimated effects of shared environmental factors on medication use for asthma at age 1 year. ETS has been more strongly associated with asthma susceptibility during early life[32] than in later childhood.[33] A recent study conducted in a hundred infants' homes with 68% prevalence of ETS demonstrated that elevated levels of particulate matter increased the odds of wheezing in the first year of life by over fourfold, and elevated levels of the nicotine metabolite cotinine in infant urine increased the odds of wheezing by over fivefold.[32] In contrast, a study of 126 children ages 6–12 years (where 68% of primary caregivers smoked in the home) showed that ETS exposure, as measured by high air nicotine and particulate matter levels, was not associated with asthma symptoms.[33] Consistent with these findings that ETS may have less effect on asthma susceptibility in later childhood compared to infancy, adjustment for ETS did not significantly alter variance component models of asthma-related outcomes at age 3 years.

Although ours is the first twin study of asthma to examine longitudinal data from infancy and early childhood, it is not the first to report age-dependent findings for heritability. A study of Danish twins ages 3–71 years demonstrated that the relative importance of environmental factors on asthma susceptibility increased over lifespan.[14] This study grouped twins into three age groups: 3–20, 20–49, and 50–71 years. Their study did not include children younger than 3 years, however, and it is possible that a strong role for shared environmental factors to asthma susceptibility would have been observed had they analyzed subjects during infancy.

Regardless of potential misclassification of transient wheeze as physician-diagnosed asthma in young twins, our findings and those of others[34], [35] support public health interventions to reduce ETS exposure to decrease asthma and viral-induced wheeze, two causes of significant morbidity in early childhood. ETS is associated with up to 12-fold higher odds of asthma-related hospitalization in children and adults.[36], [37] A study of over 21,000 hospitalizations for asthma in children demonstrated that reduction in ETS is associated with decreased asthma-related hospitalizations.[34]

The protective effect of ETS reduction on asthma morbidity is particularly relevant to individuals of Puerto Rican origin or ancestry, who not only share disproportionate burdens from asthma[18]–[20], [38] and perinatal smoking,[26] but who are also at risk for smoking cessation failure.[39] Consistent with our study, early-life ETS has been associated with asthma in Puerto Ricans.[28], [40] Previous estimates show that ETS avoidance could reduce the likelihood of severe asthma in Puerto Ricans by almost half.[35]

Our study contributes the first twin study of asthma in Hispanics to the asthma literature. Less information about asthma exists for Hispanics than for any other ethnic group in the United States, despite the fact that Hispanics are the largest and fastest growing minority group in the US and have high rates of morbidity from asthma.[41] Compared to other ethnic groups with asthma, Hispanics experience higher mortality[42] and morbidity,[43] with Puerto Ricans ranking highest. The environmental and genetic risk factors underlying asthma in Hispanics are also distinct from those for other ethnic populations.[16], [43] Therefore, twin studies of asthma in Hispanic populations, and Puerto Ricans in particular, can be highly informative and impactful, as they provide the opportunity to simultaneously examine genetic and environmental contributions to asthma in a population that suffers from asthma distinctly and disproportionately.

We recognize several limitations of our study. Firstly, we did not obtain objective measures of asthma severity or lung function. While this has been done in twin studies of older children and adults,[7], [9] it was impractical in our study participants, all of whom were younger than 4 years. To reduce the impact of diagnostic bias in young children, we analyzed not only physician-diagnosed asthma but also medication use and hospitalizations for asthma. This is reasonable in infants and young children, and preferable to sole reliance on self-reported asthma (a common approach in prior twin studies).[14] Secondly, there could be a tendency to over-diagnose or over-treat asthma in the second member of a twin pair if the first member had been diagnosed or treated for asthma. However, this should not markedly differ between pairs of MZ twins and pairs of DZ twins, and would have thus biased our results toward the null hypothesis.

Thirdly, a relatively low number of study participants had persistence of the outcomes (e.g. physician-diagnosed asthma) from age 1 year to age 3 years, and we thus had limited statistical power to examine the heritability of these sub-phenotypes. However, we had adequate power for our primary analysis of the heritability for asthma-related outcomes at age 1 year and at age 3 years. Fourthly, we lacked data on shared environmental exposures other than ETS (e.g. viral infections, allergen exposure).

## Conclusions

Among Puerto Rican children, shared environmental factors highly contribute to variance in susceptibility to physician-diagnosed asthma and need for asthma medication at age 1 year, while genetic factors contributed strongly to variation in these phenotypes at age 3 years. Early-life ETS partly explained shared environmental effects for medication use or hospitalizations for asthma at age 1 year. Early-life environmental tobacco smoke reduction could markedly reduce asthma morbidity in young Puerto Rican children.

## Supporting Information

File S1 “Supplementary Tables”.
(DOC)
