# Supplementary material for: A Twin Study of Early-Childhood Asthma in Puerto Ricans
Source: PLoS One. 2013 Jul 3;8(7):e68473. doi: 10.1371/journal.pone.0068473 (PMC3700929; doi:10.1371/journal.pone.0068473)
Supplement: File S1 — “Supplementary Tables”. (DOC) [file pone.0068473.s001.doc]

# SUPPORTING INFORMATION

A Twin Study of Early-Childhood Asthma in Puerto Ricans

Supinda Bunyavanich, MD, MPH1-5*^, Judy L. Silberg, PhD6^, Jessica Lasky-Su, Sc.D^4,5^, Nathan A. Gillespie, Ph.D^7,8^, Nancy E. Lange, MD, MPH^4,5^, Glorisa Canino, PhD^9^, Juan C. Celedόn, MD, Dr.PH^10-12^

**Table S1: Sex-stratified variance component analysis of asthma-related phenotypes at age 1 year**

| **Phenotype** | **A^#^ (95% CI)** | **C (95% CI)** | **E (95% CI)** | **P value*** |
| --- | --- | --- | --- | --- |
| Physician-diagnosed asthma |  |  |  |  |
| Males | 0.00 (0.00-0.31) | 0.98 (0.66-0.99) | 0.03 (0.00-0.13) | 0.51 |
| Females | 0.23 (0.00-0.61) | 0.75 (0.38-0.95) | 0.02 (0.00-0.18) |  |
| Asthma medication use in past 12 months |  |  |  |  |
| Males | 0.00 (0.00-0.60) | 0.94 (0.34-0.99) | 0.05 (0.00-0.25) | 0.78 |
| Females | 0.23 (0.00-0.64) | 0.74 (0.35-0.94) | 0.03 (0.00-0.19) |  |
| Hospitalized for asthma in past 12 months |  |  |  |  |
| Males | 0.35 (0.00-0.99) | 0.58 (0.00-0.98) | 0.07 (0.00-0.44) | 0.85 |
| Females | 0.97 (0.11-0.99) | 0.00 (0.00-0.69) | 0.03 (0.00-0.38) |  |

^#^A = additive genetic variance, C = shared environmental variance, E = non-shared environmental variance.

*P-value for likelihood ratio χ2 test comparing sex-stratified model allowing for different estimates in males and females to unstratified model

**Table S2: Sex-stratified v**ariance component analysis of asthma-related phenotypes at age 3 years

| **Phenotype** | **A^#^ (95% CI)** | | | **C (95% CI)** | **E (95% CI)** | | | **P value*** |  |
| --- | --- | --- | --- | --- | --- | --- | --- | --- | --- |
| Physician-diagnosed asthma | |  |  | | |  |  | | |
| Males | 0.19 (0.00-0.75) | | | 0.76 (0.21-0.96) | 0.05 (0.00-0.21) | | | 0.28 |  |
| Females | 0.80 (0.34-0.98) | | | 0.13 (0.00-0.53) | 0.06 (0.01-0.21) | | |  |  |
| Asthma Medications in past 12 months | |  |  | | |  |  | | |
| Males | 0.14 (0.00-0.58) | | | 0.83 (0.40-0.98) | 0.03 (0.00-0.15) | | | 0.12 |  |
| Females | 0.78 (0.38-0.99) | | | 0.17 (0.00-0.53) | 0.04 (0.00-0.16) | | |  |  |
| Hospitalized for asthma in past 12 months | |  |  | | |  |  | | |
| Males | 0.12 (0.00-0.97) | | | 0.78 (0.00-0.94) | 0.10 (0.01-0.38) | | | 0.52 |  |
| Females | 0.88 (0.00-0.99) | | | 0.00 (0.00-0.73) | 0.12 (0.01-0.47) | | |  |  |

^#^A = additive genetic variance, C = shared environmental variance, E = non-shared environmental variance.

*P-value for likelihood ratio χ2 test comparing sex-stratified model allowing for different estimates in males and females to unstratified model
